# Book review

**DOI:** 10.1155/S1463924686000251

**Published:** 1986

**Authors:** Andy Honeybone


### BOOK REVIEW

### Microprocessor Programming and Applications for Scientists and Engineers

By Richard R. Smardzewski (Elsevier, The Netherlands 1984), pp. 354. Hard cover. ISBN 0-444-42407-5

Written by a research chemist with those outside the electrical engineering community in mind, this first volume of Elsevier's 'Data handling in science and technology' series succeeds in being a most useful collection of information. Like many books intended for the layman, the author is forced to direct his text at an hypothetical reader assumed to be poor at mathematics but with an insatiable thirst for knowledge. Ten chapters cover bytes, diode gates, tri-state logic, the 6502 microprocessor, assembly language, analogue-to-digital converters, standard interfaces, FORTH and all the bits between. Obviously, the depth of coverage leaves questions unanswered but in general over-simplifications are avoided and the examples are practical. About one-third of the book is given over to the 6502 with practical exercises presented for the AIM 65 computer. The explanations of the processor's various addressing modes are made digestible by their interspersal with details of other operations. The 6522 versatile interface adapter chip is also covered, and the Rockwell data sheets appear in an appendix along with full 6502 instruction set details.

As a BBC microcomputer user, I find the book a constant source of reference but it must be said that with so many texts available on the subject of 6502 assembly language programming, the book could not be recommended for its coverage in this area alone. It is the additional interfacing sections which add to the value of this volume. Although the direct-set typescript lends the impression that you are leafing through someone's thesis, between the covers of this book are all the data sheets and useful pages torn from magazines that one collects over the years but never quite gets around to putting in order.

**Andy Honeybone**

### NEW JOURNAL

### Chemometrics and Intelligent Laboratory Systems

*Editor-in-Chief: D. L. Massart, Brussels, Belgium*

This new quarterly journal publishes articles about new developments on laboratory techniques in chemistry and related disciplines, which are characterized by the application of statistical and computer methods. Special attention is given to emerging new technologies and techniques for the building of intelligent laboratory systems; i.e. artificial intelligence and robotics. One of the main aims of the periodical is interdisciplinarity; more particularly it intends to build bridges between chemists, statisticians, and designers of laboratory systems. The journal deals with the following topics:

- *Chemometrics*: the chemical discipline that uses mathematical and statistical methods to design or select optimal procedures and experiments; and to provide maximum chemical information by analysing chemical data.
- Computerized acquisition, processing and evaluation of data.
- Developments in statistical theory destined to be used in chemistry.
- Intelligent laboratory systems including self-optimizing instruments and the application of expert systems and robotics in the laboratory.
- Techniques for the modelling of chemical processes such as environmental models and industrial processes including quality control.
- New software to implement the methods described above.
- Imaging techniques and graphical software applied in chemistry.

*Subscriptions cost 242 Dutch guilders or US $83.50. Requests for further information and orders may be sent to Elsevier Science Publishers, P.O. Box 330, 1000 AH Amsterdam, The Netherlands.*

